# To Keep Cider Sweet

**Published:** 1874-12

**Authors:** 


TO KEEP CIDER SWEET.—Professor Horsford, of Cambridge, recommends the following plan for preserving cider sweet:

"When the cider in the barrel is undergoing a lively fermentation, add as much white sugar as will be equal to half or three-quarters of a pound to each gallon of cider, and let the fermentation proceed until the liquid attains the right taste to suit; then add an eighth to a quarter of an ounce of sulphite (not sulphate) of lime to each gallon of cider in the cask; first mixing the powder in about a quart of the cider, and then pouring it back into the cask, and giving it a thorough shaking or rolling. After standing bunged up for a few days, for the matter added to become incorporated with the cider, it may be bottled or used from the cask."

